# Assessing the implementation fidelity of HPV self-collection offered by community health workers during home visits (the EMA strategy): a case study in a low-middle-resource setting in Argentina

**DOI:** 10.1186/s43058-022-00367-2

**Published:** 2023-01-12

**Authors:** Melisa Paolino, Victoria Sánchez Antelo, Milca Cuberli, Mariana Curotto, Anabella Le Pera, Fernando Binder, Juan David Mazzadi, Beatriz Firmenich, Silvina Arrossi

**Affiliations:** 1 Centro de Estudios de Estado y Sociedad/ Consejo Nacional de Investigaciones Científicas y Técnicas, Sánchez de Bustamante 27, 1193 Buenos Aires, Argentina; 2 Programa Nacional de Prevención de Cáncer Cervicouterino /Instituto Nacional del Cáncer (Argentina), Julio A. Roca 781, Piso 9, 1067 Buenos Aires, Argentina; 3 Centro de Estudios de Estado y Sociedad, Buenos Aires, Argentina, Sánchez de Bustamante 27, 1193 Buenos Aires, Argentina; 4 Dirección de Formación Capacitación y Planificación de Recursos Humanos en Salud. Secretaría de Salud Pública de La Matanza, Hipolito Yrigoyen 2562, 1754 Buenos Aires, San Justo Argentina

**Keywords:** Implementation fidelity; Cervical cancer prevention, HPV self-collection test, Argentina

## Abstract

**Background:**

In Argentina, HPV  self-collection offered by community health workers was demonstrated to be effective to improve cervical cancer screening uptake. Based on these findings, the EMA strategy was scaled up in nine Argentinian provinces. However, there is no evidence about the degree of fidelity—in relation to the core components proposed by the National Program on Cervical Cancer Prevention—with which this strategy was implemented in the new jurisdictions. We carried out a fidelity evaluation of the EMA strategy scaling-up aimed at evaluating the level of adherence to the core components of the EMA strategy, and how different moderating factors affected the implementation fidelity.

**Methods:**

This descriptive study used a multi-method approach involving quantitative and qualitative evaluations of the implementation fidelity using the Conceptual Framework for Implementation Fidelity. Evaluation of the degree of adherence to the core components of the EMA strategy was carried out through the analysis of a self-administered survey of health promoters, observations, and secondary data from the National Screening Information System. The analysis of moderating factors was carried out through analysis of field notes, and semi-structured interviews with key stakeholders.

**Results:**

Our results showed that the core components with highest fidelity were training, sample handling, and transportation. Regarding the offer of HPV self-collection, we found some adaptations such as locations in which health promoters offered HPV self-collection, and fewer pieces of information provided to women during the offer. In the follow-up and treatment core component, we found a reduced adherence to triage and colposcopy. Some contextual factors had a negative impact on implementation fidelity, such as urban insecurity and the reduction in the number of health promoters that offered HPV self-collection. Moderating factors that contributed to achieve high level of fidelity included a well-defined strategy with clear steps to follow, permanent feedback and high level of engagement among implementers.

**Conclusions:**

Our study shows how the analysis of fidelity and adaptations of HPV self-collection in real-world contexts are key to measure and maximize its effectiveness in low-middle-income settings.

**Supplementary Information:**

The online version contains supplementary material available at 10.1186/s43058-022-00367-2.

Contributions to the literature

- Our case study is the first that analyzed the implementation fidelity of an HPV self-collection strategy (EMA strategy) using an implementation science framework (the Conceptual Framework for Implementation Fidelity-CFIF) in a low-middle-resource setting.
- Data showed that core components of the EMA strategy were implemented with different levels of fidelity and adaptation. The use of CFIF allowed us to identify key factors that influenced the implementation process in a real-word context. Our results highlighted the need of context-adapted strategies.
- This case study provides useful evidence about implementation fidelity during a scaling-up process that is key for countries that are considering the incorporation of HPV self-collection into their cervical cancer prevention programs.

## Background

Cervical cancer is one of the leading causes of cancer death among women from low- and middle-income countries (LMIC). The disease is highly preventable with existing knowledge and technologies, and that is why the World Health Organization (WHO) has launched a global initiative to eliminate cervical cancer [1]. One key strategy to control the disease is HPV self-collection, which is effective at increasing screening uptake, especially among hard-to-reach women who are at a higher risk of this disease [2–7].

However, for HPV self-collection to have an impact in screening coverage and disease detection at the population level, it needs to be implemented in programmatic contexts.

In Argentina, HPV self-collection was implemented in the province of Jujuy as part of the EMA study (Self-collection Modality Trial, for its initials EMA in Spanish), a mixed methods study that included a cluster-randomized controlled trial to evaluate the effectiveness of HPV self-collection offered by community health workers (CHWs) at home visits to increase screening uptake [3]. The strategy resulted in a fourfold increase in screening uptake (from 20.2 to 85.9%), demonstrating that the strategy was effective to improve cervical cancer screening [3]. In 2014, EMA strategy was scaled up to the whole province [8].Evaluation of the scaling-up in Jujuy showed that the strategy resulted in a 45% increase in screening of vulnerable population, and allowed the identification of core components of the strategy, which could be implemented in new contexts. Based on these findings, the EMA strategy was scaled to other settings, and at present, nine Argentinian provinces use it to increase screening uptake among vulnerable women. The scaling-up in Argentina was carried out by the provincial programs through joint work with the team of the National Cervical Cancer Prevention Program (NCCPP). Argentina is a federal country, and the health system organization varies in each province, which can influence on how HPV self-collection is implemented locally.

One main issue of the HPV self-collection scaling-up is the implementation fidelity, this is, the degree to which an intervention is delivered as intended [9]. This analysis is especially relevant for complex interventions that consist of several core components (i.e., active ingredients that were essential to achieve the intended outcomes) [10]. Otherwise, there is a risk of evaluating effects of an intervention that has been designed as part of a randomized trial, but not fully implemented when scaled up. Maximizing fidelity to core components will be highly associated with the success in achieving change in targeted outcomes and having long-term public health impact [11]. Although there is wide evidence regarding acceptability and effectiveness of HPV self-collection in different settings, there is no evidence about the degree of fidelity with which this complex intervention is implemented in real-word contexts. To our knowledge, this is the first study to analyze fidelity of an HPV self-collection strategy using the Conceptual Framework for Implementation Fidelity (CFIF) in a low/middle-resource setting. Our results provide useful evidence about implementation fidelity during a scaling-up process that is key for countries that are considering the incorporation of HPV self-collection into their cervical cancer prevention programs.

**Fig. 1:**
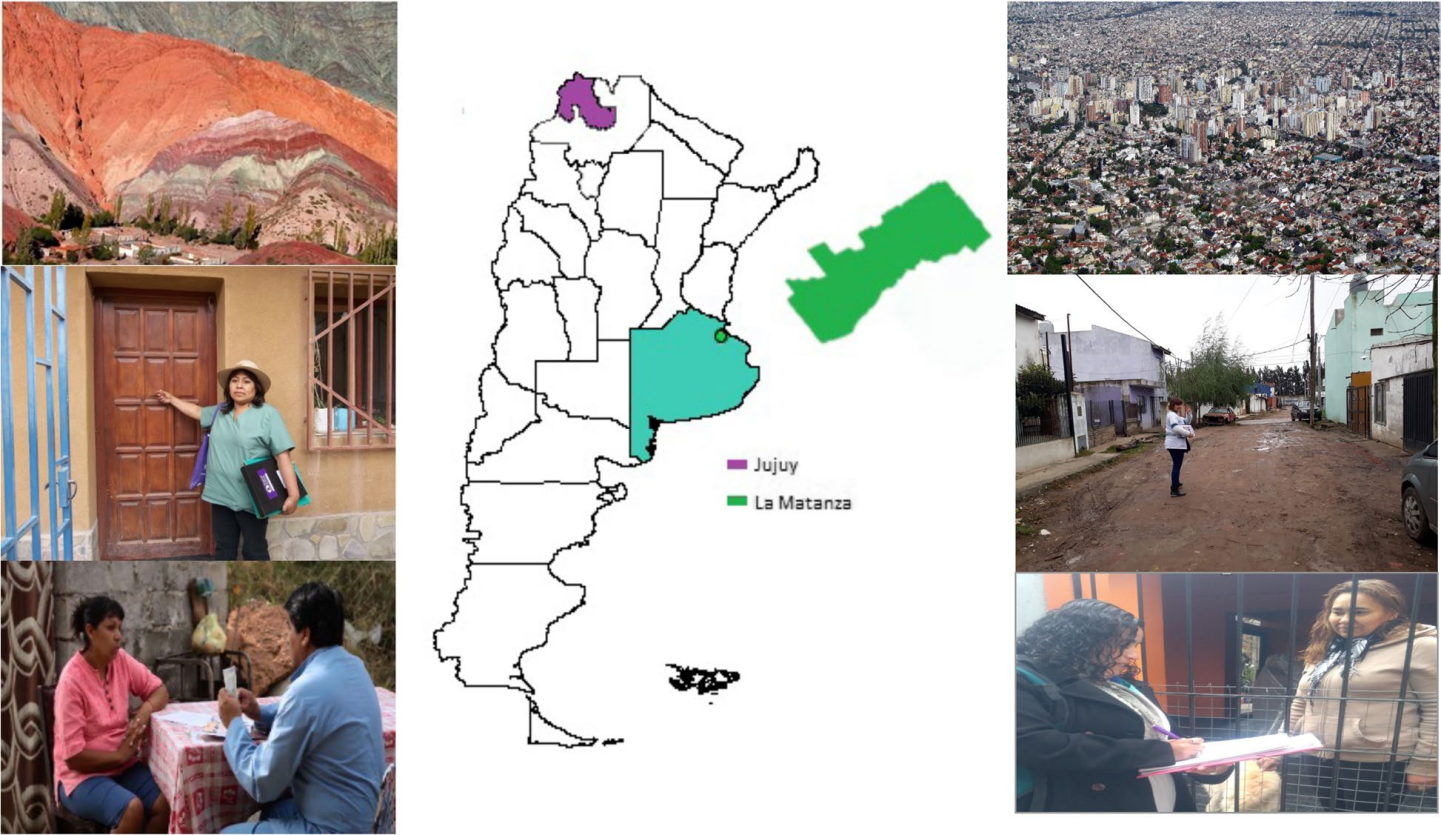
Jujuy and La Matanza settings

We present the results of an evaluation of implementation fidelity of the EMA strategy scaling-up in Argentina. The study was carried out in La Matanza, a district in the Metropolitan Area of Buenos Aires, an urban setting very different from Jujuy (Fig. 1) where the EMA study was conducted. The specific aims of the study were (1) to evaluate the level of adherence to the core components of the EMA strategy, and (2) to evaluate how different moderating factors affected the implementation fidelity. The Standards for Reporting Implementation Studies (StaRI) Statement were used to guide research reporting (Additional file 1) [12].

## Methods

### Study design

**Fig. 2:**
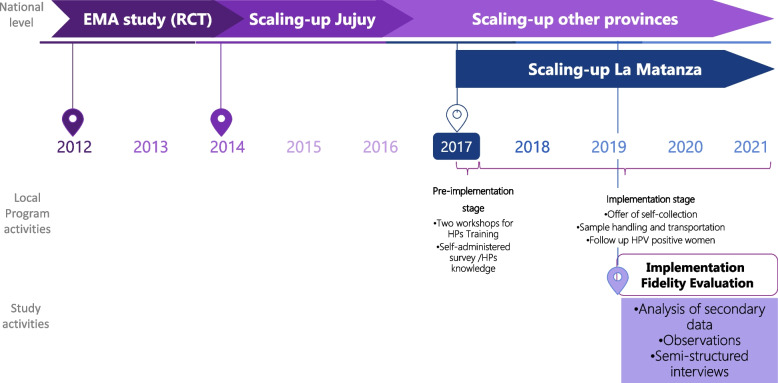
Phases of EMA strategy implementation and evaluation

We carried out a descriptive study using multiple-methods involving quantitative and qualitative evaluations of the implementation fidelity using the CFIF [9]. A multiple-method study uses both quantitative and qualitative techniques, as distinctly separate parts of one research program [13]. Evaluation of the degree of adherence to the core components of the EMA strategy was carried out through the analysis of a self-administered survey of health promoters, observations, and secondary data from the national screening information system (SITAM, for its initials in Spanish). The analysis of moderating factors was carried out through semi-structured interviews with key stakeholders, and analysis of field notes that provided complementary information on different factors that affected the fidelity of implementation. Phases of EMA strategy implementation and evaluation are showed in Fig. 2.

### Setting

The study was carried out in La Matanza, a district in the Metropolitan Area of Buenos Aires (Fig. 1). La Matanza has 2 million inhabitants, 50% of which are poor. La Matanza’s public health system comprised a network of hospitals and primary health care centers. For the uninsured population, health services are provided free of cost, including screening, diagnosis, and treatment. In La Matanza, programmatic population-based self-collection was initiated in 2017. HPV testing (Hybrid Capture 2; Qiagen, Germantown, MD, USA) was introduced as primary screening for women aged 30 and older attending the public health system. HPV self-collection is offered by health promoters during home visits. Unlike CHWs in Jujuy, health promoters did not belong to the permanent staff of the health system; they were women from the community who received social plans (conditional income transfer for protection of families in poverty conditions) provided by the Social Development National Ministry.

### Conceptual Framework for Implementation Fidelity

**Fig. 3:**
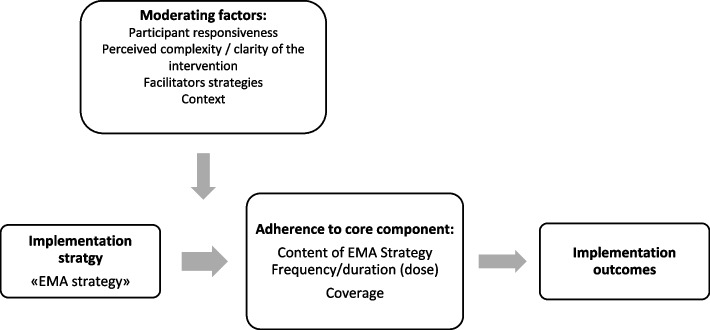
CFIF model adapted to EMA strategy. Adapted from Caroll et al., 2007 [9]; Hasson et al., 2010 [10]

The CFIF [9] was used as a conceptual model to retrospectively evaluate the implementation fidelity of EMA strategy. We chose the CFIF because it is particularly appropriate to evaluate implementation fidelity of complex interventions—as the EMA strategy—because it allows a comprehensive assessment of different dimensions of implementation fidelity and the moderating factors that may influence it (Fig. 3). This model was integrated in all stages of the research, including conceptualization (e.g., selecting implementation components on which to focus), data collection (e.g., using components of the conceptual model to design interview guides), and analysis.

Several terms have been used interchangeably with fidelity in the literature, including treatment fidelity [14–17], intervention fidelity [18, 19], implementation fidelity [9, 10], or fidelity of implementation [20]. This variety in terms associated with fidelity is related to the fact that initially, fidelity was mostly referred to the evaluation of what was delivered clinically in randomized trials. At present, it applies to all types of studies, from tightly controlled efficacy trials to implementation studies, although the focus on fidelity varies across each type of study [11]. Following Carroll et al. [9], we have chosen the term “implementation fidelity” defined as the degree to which the EMA strategy was implemented as it was intended in the original model. It relates to the process of implementation of core strategy components [21]. Implementation fidelity is critical to a successful translation of evidence-based strategies into real contexts, and it is a factor that may influence the relationship between the strategy and its intended outcomes. Evaluation of implementation fidelity is particularly important given the greater potential for inconsistencies in implementation of a strategy in the real world rather than in experimental conditions [9].

For Carroll et al. [9], the measurement of implementation fidelity is the measurement of adherence. Adherence includes the subcategories of content (i.e., “active ingredients”), frequency, duration, and coverage (i.e., dose). The *content* of the intervention may be seen as its “active ingredients” or components that are essential to achieve the indented outcomes. In our case study, the content was defined as the core components of the EMA strategy (which are described below). *Coverage* refers to the degree to which women who met inclusion criteria accepted the intervention, and *frequency and duration* refer to whether the intervention/strategy was delivered with the regularity and duration planned by its designers. In addition, the level of implementation fidelity achieved may be influenced by other variables called moderating factors. The original model described four moderating factors: intervention complexity, facilitation strategies, quality of delivery, and participant responsiveness. The CFIF model used in this study has been modified based on the adaptation made by Hasson et al. [10, 22] for the assessment of fidelity of complex interventions. Following Hasson’s model, we have included the following moderating factors:

- *Participant responsiveness* refers to how well participants respond to or are engaged by the intervention. In our case study, responsiveness refers to the engagement of health staff involved in EMA strategy implementation.
- *Intervention complexity* refers both to intervention and its implementation strategy characteristics—e.g., number of core components—and to the way in which they have been described and transmitted to the implementers [9].
- *Facilitation strategies* refers to support strategies that may be used both to optimize and to standardize implementation fidelity, i.e., to ensure that everyone is receiving the same training with the aim of the delivery of the intervention to be as uniform as possible (e.g., manuals, monitoring and feedback) [9].
- *Context* refers to the surrounding social systems such as structures and the culture of the organizations and groups, inter-organizational linkages, and historical as well as concurrent events [10, 14].

The CFIF states that different moderating factors might affect, positively or negatively, the implementation process and its fidelity. In addition, factors interact with each other, and the effect of one factor on fidelity might be influenced by another moderating factor [9].

### Description of the EMA strategy

**Fig. 4:**
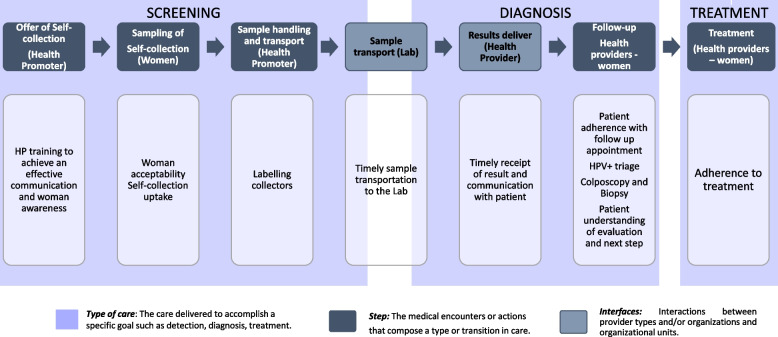
Steps and interfaces in EMA strategy. Adapted from Taplin and Rodgers 2010 [26] and Nekhlyudov et al., 2010 [27].

The EMA strategy is a complex evidence-based intervention that was implemented, evaluated and scaled up nested into the Jujuy Demonstration Project (JDP) [23]—more details about the EMA strategy can be found elsewhere [3, 8]. It consists of several active ingredients that address multifaceted processes within interpersonal, organizational, and community contexts [24, 25]. It is based on the concept of Cancer Care Continuum defined as a process of care consisting of several steps (screening-diagnosis-treatment) and interfaces between patients, providers, and organizations. Central to the process of care across the continuum is the transfer of information and responsibility from one institution to another, from one health professional to another, and from providers to patients [26]. The EMA strategy involves door-to-door offer of HPV self-collection to women by trained health staff together with provision of information about how to perform self-collection, sampling handling, and transportation of samples to the HPV laboratory, follow-up, and treatment of HPV-positive women at health centers (Fig. 4) [8]. These components are essential to achieve high level of adherence to screening, triage, diagnosis, and treatment, the necessary steps to assure the screening program effectiveness to prevent cervical cancer. CFIF dimensions applied to the EMA strategy are as follows:

#### Content

As mentioned above, the EMA strategy included four core components that were defined as the content of the intervention. Table 1 shows specifications for each core component following Proctor et al. model [25].

**Table 1:** Specification of core component of EMA strategy following Proctor model [25]

**Core component: training**	
Actor	National and Local Training team experts in Cervical Cancer Prevention, HPV test and Health Communication. Experts that participated in EMA Study (RCT and Scaling Up in Jujuy province).
Actions	To provide training through different techniques (with experts´ presentations, discussions in small groups, and role playing) regarding: cervical cancer prevention and HPV, HPV results. To evaluate knowledge acquired during the training (self-administered survey)
Target of the action	Health promoters in charge of the offer of self-collection, health supervisors, health professionals involved in EMA strategy implementation in La Matanza.
Temporality	Pre-implementation phase
Dose	Two workshops
Implementation outcome	Adoption and Fidelity
Justification	Health education/training of implementers
**Core component: offer of self-collection**	
Actor	Trained health promoters
Actions	To identify the target population: age, pregnancy, previous screening. To provide information about HPV test and cervical cancer To explain on how to perform self-collection
Target of the action	Women aged 30 and over
Temporality	Implementation phase
Dose	10 min step-by-step explanation of how to do self-collection
Implementation outcome	Acceptability and Fidelity
Justification	Door to door offer is effective to increase screening uptake and acceptability of HPV self-collection [3–6].
**Core component: sample handling and transportation**	
Actor	Health promoters who offer self-collection /supervisors/ health professionals involved in cervical cancer prevention program
Actions	To label collectors with the woman’s name and the national unique identification number. To fill in HPV forms. To transport samples at room temperature to primary health care centers. To assure that specimens arrive to HPV laboratory within 14 days after sample collection.
Target of the action	Women aged 30+ who performed HPV self-collection
Temporality	Implementation phase: Labelling collector: during self-collection offer. Transportation: within 14 days after sample collection.
Dose	Always
Implementation outcome	Fidelity
Justification	Protocol of identification and transportation of samples following manufacturer’s instructions.
**Core component: follow-up and treatment**	
Actor	Health professionals in charge of triage Pap in primary health centers (e.g., gynecologist, obstetricians, nurses), Gynecologist in charge of colposcopy and treatment (second and third-level hospitals)
Actions	Follow-up and treatment should be organized according to national guidelines [13]: (1) HPV-positive women should be referred to cytology triage. HPV-positive women with normal cytology should repeat the HPV test in 18 months. (2) Women with abnormal cytology (atypical squamous cells of undetermined significance; atypical cells cannot rule out high-grade squamous intraepithelial lesion; high-grade squamous intraepithelial lesions; and cancer) should be referred to colposcopy, and biopsy if needed. (3) Biopsies should be reported according to cervical intraepithelial neoplasia (CIN) terminology. Identified cases of CIN2+ should be treated according to standard protocols (loop electrosurgical excision procedure (LEEP) for CIN2+) and (4) HPV-negative women should be advised to repeat screening in 5 years.
Target of the action	HPV-positive women
Temporality	Implementation phase: after an HPV-positive results
Dose	Always
Implementation outcome	Fidelity
Justification	Adherence to triage, diagnosis and treatment are essential to reduce cervical cancer incidence and mortality

##### Training

Self-collection should be offered by trained health promoters. Training should include at least (1) two workshops about cervical cancer prevention and HPV test, with experts’ presentations, discussions in small groups, and role playing to recreate different scenarios during the offer (Table 2), and (2) at the end of the last workshop, inviting health promoters to complete a self-administered survey to evaluate knowledge acquired during training.

**Table 2:** Description of training workshops

Sections	Description
Project background	Provides information about the EMA study and the scaling-up into the programmatic context.
Cervical cancer	Provides scientific information about cervical cancer and its relation with HPV.
HPV testing	Provides basic information regarding HPV testing as primary screening for cervical cancer prevention.
HPV self-collection	Provides specific information about HPV self-collection: differences with clinician-collected tests, steps of self-collection take-up, understanding results of self-collection, and follow-up of HPV+ women.
Communication skills	Provides communication skills to conduct the educational talk (instruct women on how to perform self-collection).
Logistical procedures	Training about how to label and transport samples.

Adapted from Arrossi et al., 2017 [8].

##### Offer of self-collection

Self-collection offered during home visits by trained health promoters should include at least (1) identifying the target population, (2) offering of self-collection, (3) providing women with information about cervical cancer prevention and HPV self-collection (Table 3), (4) offering women to perform HPV self-collection, and a 10-min step-by-step explanation on how to perform it using communication support material (Additional file 2), and (5) providing women with the HPV collector to collect the sample.

**Table 3:** Description of core components, sub-dimension, items, and activities included in the checklist

**Core component: offer of self-collection**	
Sub-dimension: Identification of target population (3 items)	
*Age*	
*Pregnancy*	
*Previous HPV screening*	
Sub-dimension: key information during the self-collection offer (7 items)	
*Information about HPV test*	
*Information about cervical cancer prevention*	
*Information about self-collection*	
*Information about self-collection is painless*	
*Information about possible results (HPV positive/negative)*	
*Information about results delivery date*	
*Information about results delivery methods*	
Sub-dimension: step-by-step explanation on how to perform self-collection (6 items)	
*Private place to perform self-collection*	
*Information about different positions to perform self-collection*	
*Warning about the liquid inside the tube*	
*Explanation about how to insert the brush*	
*Explanation about how to rotate the brush 3 times*	
*Use of communication support material*	
**Core component: sample handling and transportation (4 activities)**	
*To check that the tube was correctly closed*	
*To carry the tube in vertical position*	
*To label collectors*	
*To fill the HPV form*	

##### Sample handling and transportation

Sample handling and transportation should include at least (1) labelling collectors with the woman’s name and the national unique identification number, (2) filling in sample collection forms, (3) transporting the samples at room temperature to primary health care centers and then to the HPV laboratory, (4) assuring that specimens arrive at the HPV laboratory within 14 days after sample collection, and (5) discarding samples without liquid, brush, or identification data at the HPV laboratory.

##### Follow-up and treatment

Follow-up and treatment should be organized according to national guidelines [28]: (1) HPV-positive women should be referred to cytology triage. HPV-positive women with normal cytology should repeat the HPV test in 18 months, (2) women with abnormal cytology (ASCUS+) should be referred to colposcopy, and biopsy if needed, (3) identified cases of CIN2+ should be treated according to standard protocols (loop electrosurgical excision procedure (LEEP) or conization, and (4) HPV-negative women should be repeat screening in 5 years.

#### Dose (duration)

The “dose” of EMA strategy was defined based on the expected duration of the step-by-step explanation on how to perform self-collection (10 min).

#### Coverage (acceptability)

Coverage was defined as the proportion of women who accepted self-collection after the offer of self-collection. The expected acceptability of self-collection was based on results of the EMA study (86%).

Indicators and sources of data used to measure adherence (content, dose, and coverage) to the core components of the EMA strategy are presented in Table 4.

**Table 4:** Adherence to core component of EMA strategy: indicators and source of data

Adherence	Sub-dimensions	Indicators	Source of data
**Content: core components of the strategy**			
**Training**	Training	Number of planned workshops implemented	Training registries
		Inclusion of experts’ presentations, role playing and discussions in small groups	Training material
	Participation in training	% of health promoters that participated in the workshops	Training registries
	Level of knowledge about the strategy among health promoters	% of health promoters with adequate knowledge about EMA strategy	Self-administered questionnaires after training
**Offer of HPV self-collection**	**Place of offering SC**	% of self-collection offered during home visit	Checklist
	***Identification of target population***		
	Age	% of self-collection offers in which health promoter asked the woman’s age	Checklist
	Pregnancy	% of self-collection offers which HPs asked if the woman was pregnant	Checklist
	Previous screening	% of SC offers in which HPs asked if the woman had had a previous HPV test	Checklist
	**Key information during SC offer**		
	Information about HPV test	% of SC offers in which HPs mentioned information about HPV test	Checklist
	Information about cervical cancer prevention	% of SC offers in which HPs mentioned information about cervical cancer prevention	Checklist
	Information about HPV self-collection	% of SC offers in which HPs mentioned information about self-collection	Checklist
	Information about self-collection is painless	% of SC offers in which HPs mentioned that self-collection was painless	Checklist
	Information about possible results (HPV positive/negative)	% of SC offers in which HPs mentioned information about possible HPV results	Checklist
	Information about results delivery date	% of SC offers in which HPs mentioned information about results delivery date	Checklist
	Information about results delivery methods	% of SC offers in which HPs mentioned information about results delivery methods	Checklist
	**Explanation on how to perform self-collection**		
	Private place to perform SC	% of SC offers in which HPs suggested a private place to perform self-collection	Checklist
	Information about different positions to perform self-collection	% of SC offers in which HPs mentioned different positions in which to perform SC.	Checklist
	Warning about the liquid inside the tube	% of SC offers in which HPs warned the woman to be careful with the liquid inside the tube	Checklist
	Explanation about how to insert the brush	% of SC offers in which HPs explained the woman that she had to insert the brush into her vagina until it reached the top	Checklist
	Explanation about how to rotate the brush 3 times	% of SC offers in which HPs explained the woman that she had to rotate the brush 3 times.	Checklist
	Use of communication support material	% of SC offers in which HPs used communication support material	Checklist
**Sample handling and transportation**	**Sampling handling and transportation protocol (among women who accept SC,** ***n*=50)**		
	To check that the tube was correctly closed	% of SC offers in which HPs checked that the tube was correctly closed	Checklist
	To transport the tube in vertical position	% of SC offers in which HPs transported the tube in vertical position	Checklist
	To label collectors	% of SC offers in which HPs labeled collectors	Checklist
	To fill the HPV form	% of SC offers in which HPs filled the HPV form	Checklist
**Follow-up and treatment**	Adherence to triage	% of HPV-positive women with triage Pap in 120 days	SITAM
	Adherence to diagnosis	% of HPV-positive/abnormal Pap women with colposcopy	SITAM
	Adherence to treatment	% of women with CIN2+ with treatment	SITAM
**Dose**			
Duration	Duration of SC offers	Mean duration of SC offers	Checklist
**Coverage**			
Coverage	Acceptability	% of eligible women who accepted to perform self-collection	Checklist

### Data collection

#### Quantitative data sources

##### Observations

To evaluate the implementation fidelity of the core components “offer of self-collection” and “sample handling and transportation,” we conducted observations with limited participation during routine self-collection offers. Between June 14 and July 23, 2019, four female trained observers carried out 74 observations. All the health promoters of La Matanza who received training in 2017 and offered self-collection in 2019 were eligible to participate. In total, 78 health promoters who were working in the PHC system were eligible. Using a computer-generated random number list, we selected a sample of 20 health promoters to be accompanied during a workday. An observation protocol (checklist) was developed based on the competencies and activities carried out by the health promoters. We first identified a comprehensive list of planned activities by reviewing programmatic documents and training materials. We then classified this list of activities according to the content of the strategy (Table 3). The list of activities was validated by the training coordinator of the NPCCP. For each planned activity, we evaluated whether it was implemented according to the EMA model (Yes/No). In addition, observers registered any adaptation of the offer.

##### Self-administered questionnaires

An ad hoc self-administered survey was developed to evaluate knowledge acquired by health promoters during the training workshops. At the end of the last workshop, health promoters were asked to complete an anonymous, self-administered survey. In total 171 health promoters completed the survey. This survey included 11 questions (multiple choice) regarding scientific data on cervical cancer and its relationship with HPV, basic information on HPV testing, the step-by-step offer of self-collection, and the follow-up of HPV+ women. Multiple choice options included only one correct answer. Results were registered in a specific database for processing and analysis.

##### National screening information system (SITAM)

We used data extracted from SITAM to evaluate the core component “follow-up and treatment” We analyzed the SITAM database containing records of all women aged 30 years and older screened in La Matanza using HPV self-collection during 2017–2018, and data recorded until December 2019 for the follow-up. Colposcopies, biopsies, and treatments not registered in SITAM were considered lost to follow-up. The data were accessed by authorized healthcare workers and researchers.

#### Qualitative data sources

During April–June 2020, we carried out six semi-structured interviews with key informants to explore their views on the EMA strategy and moderating factors that affect its implementation. We selected key informants who had been involved in the implementation of the self-collection strategy since it was introduced in 2017 and played a central role in different stages of the process. Interviews were conducted in Spanish by a female social science researcher, specialist in qualitative research (VSA). She did not live in La Matanza and did not have any relation whatsoever with the health care facilities or their authorities. Due to the COVID-19 pandemic, these interviews were carried out online. Online tools for data collection are suitable for different topics and allowed us to solve logistical issues [29]. In addition, we analyzed field notes that were taken during observations, and training materials (e.g., training documents).

### Data analysis

#### Quantitative data

Indicators were analyzed through frequencies and percentages. The percentage of implemented activities was calculated as follows: Total number of implemented activities /total number of planned activities following EMA model*100. The percentage of implemented activities represented the degree of fidelity. Based on other studies that evaluate implementation fidelity in community settings [30], the following scoring categories were used in this study: 80–100%, high; 79–51%, moderate; and ≤ 50%, low.

We also calculated the percentage of adequate knowledge, defined as percentage of correct answers in the self-administered survey as follows: number of correct answers / total number of answers (adequate knowledge >70% correct answers).

#### Qualitative data

Qualitative data were analyzed thematically [31] using the CFIF dimensions. Two researchers became independently familiar with the data through audios and transcriptions and classified data using an initial codebook, to later compare and generate themes [32], debate, and resolve the inconsistencies with the other team members. We sought quotation examples that adequately graphed each theme and were the most relevant to assess implementation fidelity. Atlas.Ti (version 7.5.4, ATLAS.ti Scientific Software Development GmbH, Berlin) was used for data processing.

### Stakeholder engagement

Municipal health authorities and professionals actively participated in the planning, implementation, and fidelity evaluation of the strategy. The Director of Training of the Primary Health Care Direction of La Matanza, who was a co-researcher, participated in the design, implementation, and data analysis. All the staff of the Secretariat of Health helped to organize the field work and participated in meetings where several aspects of the project were discussed. The Secretary of Health of La Matanza, health authority in charge of the primary health care centers, gave his support to the project. In addition, NPCCP staff participated in the design, checklist elaboration, field work, and data analysis. Besides, Argentina NCI funded the study.

## Results

### Adherence to the core components of EMA strategy

**Fig. 5:**
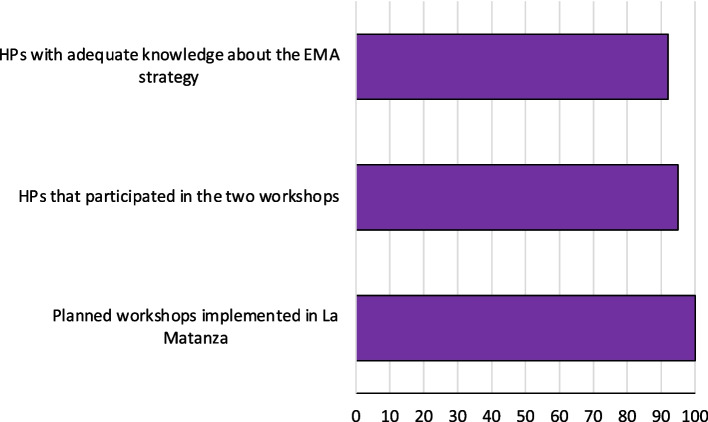
Core component: training. HPs: health promoters

**Fig. 6:**
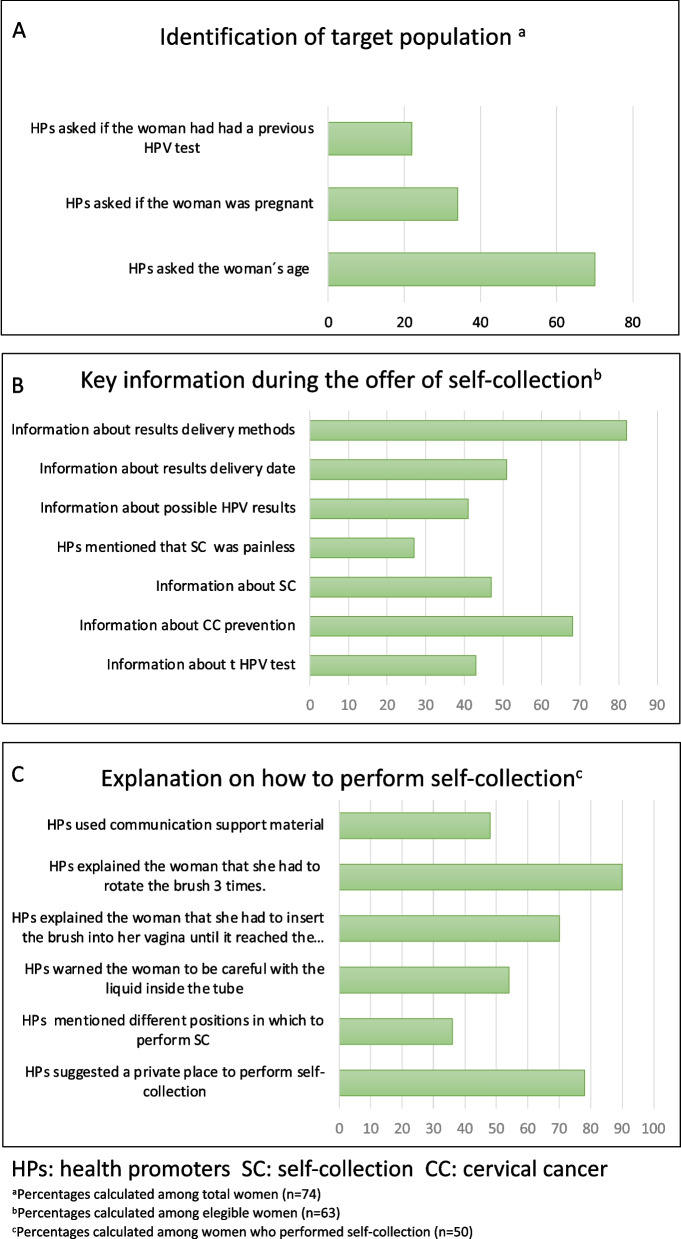
Core component: offer of self-collection

**Fig. 7:**
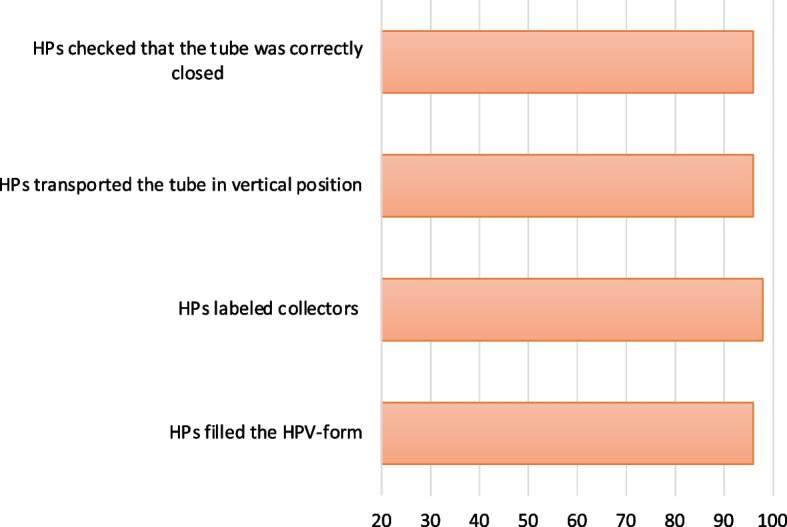
Core component: sample handling and transportation. HPs: health promoters

The degree of adherence to activities included in the four core components of the EMA strategy is shown in Table 5 and Figs. 5, 6, and 7.

**Table 5:** Adherence to the core components of EMA strategy and moderating factors

Adherence	Level of adherence (% range of adherence)	Moderating factors that affect level of adherence (+ positive / − negative)
**Content: core components of the strategy**		
**Training**	92–100%	**Context:** Stakeholder engagement, Political will (+)
		**Political will (+) Participant responsiveness:** motivation of health promoters, active participation of local stakeholders during planning and implementation of training (pre-implementation phase) (+)
**Offer of HPV self-collection**	54%	**Context:** urban insecurity (−) / reduction of number of health promoters that were involved in cervical cancer prevention (−) Previous experience in community work allowed changes in the place of the offer (+)
	22–70%	**Context:** less time to do the offer (−) /reduction of information during the offer **Intervention complexity**: several pieces of information (-)
	27–82%	
	36–90%	
**Sample handling and transportation**	96–98%	**Facilitator strategies: f**eedback and supervision in different levels of the health system: health care centers—HPV lab—second-level hospitals (+)
		**Participant responsiveness:** motivation and active participation of health promoters, supervisors, and local stakeholders (+)
**Follow-up and treatment**	Triage: 38% (318/830)	**Intervention complexity:** different levels of health system involved in screening—triage—diagnosis and treatment (−)
	Diagnosis: 64% (34/49)	
	Treatment: 100% (13/13)	
**Dose**		
Duration (mean)	8 min	
**Coverage**		
Coverage (% of women who accepted SC)	79%

#### Content

##### Training

Training was implemented with high level of adherence. In 2017, national and municipal team members led two workshops (100% of planned workshops). These teams planned and carried out the workshops following the EMA strategy model, which included experts’ presentation, role playing, and discussions in small groups. In total, 95% of health promoters participated in both workshops. In the last workshop, acquired knowledge was evaluated, 91% of health promoters had adequate knowledge about the strategy (Table 5, Fig. 5).

##### Offer of self-collection

This core component was implemented with adaptations. Percentages of adherence among its 16 items were highly variable (from 22 to 82%). Fifty-four percent of self-collection offers were carried out during home visits and 46% were carried out in community health meetings and waiting rooms in primary health care centers. Regarding “Identification of target population,” most health promoters (70%) asked women their age; however, previous HPV testing and pregnancy status were asked less frequently; in some cases, health promoters evaluated the eligibility status without asking women specific information. Observations of information provided about cervical cancer prevention and HPV self-collection showed that in 68 and 82% of self-collection offers, health promoters mentioned the relationship between the test and cervical cancer prevention and the result-delivery methods, respectively. However, information about the HPV test characteristics (43%), self-collection (27%), and HPV results (41%) was provided to a lesser extent. Four out of the six items included in the “step-by-step explanation about how to perform self-collection” were mentioned in more than 54% of observations (range 54–90%). Communication support materials were used in 48% of the offers (Table 5, Fig. 6).

##### Sample handling and transportation

All activities included in this component were performed with high level of adherence: range 96–98% of observations (Table 5 and Fig. 7).

##### Follow-up and treatment

Between May 2017 and December 2018, 5069 women aged 30 and older were screened with self-collection (51% of total HPV-tested women, *n* = 5069/9977). Among them, 16% were HPV positive. Thirty-eight percent of HPV-positive women had a triage test at 120 days after screening showing a low level of adherence. Adherence to diagnosis and treatment were higher: 64% of women with positive triage performed a colposcopy. In total, 13 CIN2+ were identified and 100% were treated.

#### Dose

In general, health promoters spent less time than stipulated to offer self-collection (mean 8 min; range 1–17 min); therefore, as we mentioned above, fewer pieces of information were provided.

#### Coverage

During the study, 63 of 74 visited women were considered eligible to perform self-collection. Among them, 79% accepted to perform it.

### Moderating factors

#### Participant responsiveness

We found a high level of engagement among all actors involved in the implementation. Decision makers from the three levels (national, provincial, municipal) were involved since the beginning, with participation in round tables organized by the NPCC to plan the EMA strategy implementation at the local level. As mentioned by one of the interviewees, this involvement of all governmental level was key to integrate and coordinate all the necessary activities:

It requires a lot of will at the level of health policy. I mean, not only from the person who is carrying it out... In other words, it seems to me that this worked at least in [La] Matanza because there were people to make it work in the municipality, and in the nation and in the province, in other words, the three of them. Not from just one because just one can’t achieve this.

(E1. Training Coordinator)

Additionally, all interviewees recognized the active involvement of health professionals, HPV laboratory staff, and the health promoters. This involvement was related to the EMA strategy, which was considered an innovative and important tool to facilitate the health system to reach a population that is usually out of reach. In agreement with this recognition, data from field notes showed high acceptability and motivation among health promoters and their supervisors.

The self-collection changed everything... because sometimes many women were reluctant to go to the gynecologist. This was revolutionary, this was bringing health into their homes... so innovative...

(E3. Supervisor of Primary Health Care Direction)

... Today, I can’t imagine working on cervical cancer prevention without the HPV test. I would say it would be like taking 25 steps backwards (...) I think the HPV test and the whole [EMA] strategy to ensure women’s access is fundamental. We are fully confident that this is the way forward.

(E2. Navigator)

#### Perceived complexity

The EMA strategy was defined by the interviewees as a complex intervention due to the several core components that included and the several health system institutions and providers that needed to be involved:

I think it’s a complex process that requires many actors and each of them must carry out their part correctly, because if someone takes the sample and doesn’t store it properly or if when they go to offer it, they don’t explain it well… There are many participants, and they all have to be linked together, it’s a gear…

(E1. Municipal Training Coordinator)

[The most complex issue is] the relation with the second-level hospital, to continue with triage and treatment. But this was worked on and greatly improved.

(E3. Supervisor of Primary Health Care Direction)

In spite of its perceived complexity, stakeholders characterized the EMA strategy as well-planned. All agreed in defining the core components as clear stages to follow, and they mentioned that they had clear and sufficient information on how to proceed during the implementation. The interviewees highlighted the relevance of the training, and they described it as balanced between the use of accurate medical terms and accessible language to laypersons.

[The strategy] isn’t too complex. The key part of it is training the health personnel. Once they’re involved and trained, it’s very easy.

[The training content] was perfect according to the target audience, it was very well thought out: they didn’t talk in technicalities, they went straight to the point but not as if they were children. Everything was said with the correct terminology, but with simple and easy explanations...

(E1. Municipal Training coordinator)

#### Facilitator strategies

A key facilitation strategy was the permanent feedback among implementers (e.g., the laboratory team, the navigator, and the supervisors). As the implementation progressed, this allowed them to identify problems in sample handling, transportation, or data registration and therefore facilitate their resolution. These activities affected positively implementation fidelity. In fact, sample handling and transportation was one of the core components with high level of fidelity (Table 5, Fig. 7).

We held a meeting with all the supervisors and health promoters on sample issues, as sometimes the samples arrived without the forms and in the meeting, we said, ‘But a key person is missing here, the driver who carries the samples is missing’. So, we met with the driver to explain.

(E4. Supervisor of Primary Health Care Direction)

In addition, training organization and education materials were also considered facilitators of the strategy: for example, the meetings held during the pre-implementation phase to plan training activities and adapt training materials to local needs, as well as the use of handbooks and flyers to guide and standardize the delivery of the intervention.

[The health promoters] received explanations on how to write the data in clear handwriting... everything was explained. They were given a flip chart with an explanation to be provided to the women, as well as handouts. They could keep the flipchart and that could help them explain everything to the women...everything they had learned....

(E1. Municipal Training Coordinator)

#### Context

Some contextual factors had a direct impact on implementation fidelity. As above mentioned, health promoters did not belong to the permanent staff of the health system. This fact sometimes produced weak linkage with the formal health system and unstable or unsustainable financing. For example, financial resources for the employment of health promoters fluctuated during the implementation phase. In 2019, there was a change in the national Social Development Ministry policy of health promoters. This change resulted in a decrease of the number of health promoters, which was reduced from 171 in 2017 to 78 in 2019.

In addition, social insecurity negatively affected implementation fidelity. La Matanza is a complex urban setting. It is a large territory, with social inequality and with high levels of insecurity (i.e., perception of risk as well as lack of safety because of a high level of crime in some suburban areas). Data from semi-structure interviews and field notes showed that insecurity was an important moderator of the implementation (in fact, one of the observers had to interrupt field work due to a shooting incident).

[when we were not working in our usual neighborhood] It was very difficult to go into a house because of the safety issue. You know that not everyone opens the door of their house...

(E1. Municipal Training Coordinator)

Due to insecurity problems and the reduction of health promoters, local health staff decided to offer HPV self-collection during community activities carried out in schools, churches, fairs, and squares. This was possible because of the presence of a motivated health system staff that understood the context and that had a broad experience in primary health care and community work. In addition, the municipality of La Matanza had a health trailer that was used to provide women with a place to perform HPV self-collection when offered at community places. Interviewees evaluated this adaptation as a satisfactory response to guarantee the intervention.

In addition to the door-to-door offer... we implemented what were called ‘healthy circuits’ which is where the mobile hospital went. This was a trailer that had several clinics, where they worked on other issues such as diabetes, hypertension... And there the health promoters offered the self-collection test... That same moment, in the trailer bathroom, they would take the self-collection test.

(E3_Supervisor of Primary Health Care Direction)

## Discussion

This is, to our knowledge, the first study that analyzed implementation fidelity of an HPV self-collection strategy using implementation science methods. Utilization of the CFIF allowed us to carry out a comprehensive evaluation of adherence to core components of the EMA strategy and to identify key factors that influenced its implementation. Using a multi-method approach, we analyzed the implementation fidelity of the EMA strategy in La Matanza, an urban and low-middle-resource setting in Buenos Aires, a different context from Jujuy, in which EMA strategy was designed. Our results showed that the core components with highest fidelity were training and sample handling and transportation. Regarding the offer of self-collection, we found some adaptations such as the locations in which health promoters offered self-collection, and fewer pieces of information provided to women. In follow-up and treatment, we found a reduced adherence to triage. Some contextual factors had a negative impact on implementation fidelity, such as insecurity and the reduction in the number of health promoters that offered self-collection. Moderating factors that contributed to achieve high level of fidelity included a well-defined strategy with clear steps to follow, permanent feedback, and high level of engagement among implementers.

Our results showed that training was implemented with high level of fidelity. The provision of supportive materials for training may have a direct impact on the quality with which the strategy is delivered, and this may, in turn, affect the fidelity with which a strategy is implemented [9]. Key stakeholders mentioned that training organization and education materials were facilitators of the strategy. Similar results were found in Jujuy, where CHWs mentioned that training organization was the main facilitator of the implementation of EMA strategy [33]. Training is a key core component that allows standardization of complex interventions and should be considered as a priority core component in future scaling-up of the EMA strategy.

The core component “offer of self-collection” was implemented with adaptations: collective rather than individual offer and change of the place where it is offered. These adaptations were due to context factors: urban context, insecurity, and the increase in the number of women to be visited by each health promoter after cuts in social plans resulted in less health promoters available to offer self-collection. These adaptations emerged during the implementation process and were proposed by health staff with high motivation and engagement. This result is similar to the result reported by Hassson et al. [10, 22]. In their study they observed that staff enthusiasm for the project (responsiveness) was high, and this seemed to be a reason for adapting components to the intervention [22]. Wide evidence illustrates how adaptation of evidence-based interventions is the rule rather than the exception when used in real-world practice [34]. Although there are studies showing high fidelity to be related to better outcomes than low fidelity [15, 17], other studies suggest that adapted interventions may be more effective than non-adapted ones [35]. As Carroll et al. mentioned, the evaluation of implementation fidelity provides a scope for identifying adaptability to local conditions. The adaptations observed during our case study could inform future scaling-up of EMA strategy in other urban settings with a dearth of human resources for the offer of self-collection.

Our results also showed that health promoters spent less time during the offer (dose), and consequently, the women received fewer pieces of information, especially regarding HPV testing and its role in cervical cancer prevention and results delivery and how to follow-up if positive. Our results suggest that these adaptations did not affect the acceptability of HPV self-collection among women, but they might have had an impact on adherence to triage and follow-up (a core component in which we observed low level of fidelity). In addition, not fully understanding the role of self-collection in cervical cancer prevention might reduce women capacity to make an informed decision. Strategies to support women with all the information included in the offer of self-collection core component might be needed to guarantee this, e.g., an app aimed at providing information and counseling to HPV+ women is being developed [36] and it might contribute to complement the explanation provided by health promoters.

HPV testing is only effective if all the women receive the corresponding follow-up and treatment. In our study, the adherence to triage was 38% at 120 days. Limited compliance to triage has been reported in Argentina [8, 23, 37, 38] and other countries [39–41]. Studies that analyzed why screen-positive women failed to complete follow-up found that in most cases it was due to problems related to the health service organization [42, 43] (i.e., delays or failures in result delivery, lack of appropriate guidance concerning the steps to follow after receiving a positive test). The EMA strategy is a complex intervention consisting of several steps that address multifaceted processes within interpersonal, organizational, and community contexts that may affect implementation fidelity. For example, if self-collection is offered during community meetings the delivery of results and referring patients for follow-up is challenging. The use of mHealth technologies to send reminders to women could be a feasible tool to reduce the time from screening to triage and improve patient-provider communication. In Jujuy, the ATICA project [44], an effectiveness-implementation hybrid type I trial, showed that a multi-component mHealth intervention increased adherence to triage among women with HPV+ self-collected tests. This strategy could be scaled up to improve the effectiveness of self-collection and reduce cervical cancer incidence and mortality.

Participant responsiveness refers to how well participants are engaged by an intervention/strategy [9, 22]. Several studies have shown that higher levels of implementation fidelity can be achieved when those responsible for implementing a strategy are engaged [9, 10]. In our study, participant engagement was high, with active participation of health authorities during design and implementation of self-collection, and high acceptability of the strategy by health promoters. Joint ownership and design of interventions has been pointed out as a facilitator strategy that contributes to increase motivation and engagement of health staff [45, 46]. Participant responsiveness also includes judgments by participants about the outcomes and relevance of an intervention [9]. In our study, both stakeholders and health promoters considered self-collection as a tool that allowed the health system to reach a population that was usually unreachable and, therefore, have an impact in the burden of the disease. Feedback is also a key facilitator of participant responsiveness [9]. In our study, permanent feedback among the laboratory team, the navigator, and the supervisors contributed to identify and solve problems related to sample handling and transportation and therefore achieve a high level of fidelity. Evidence has shown that a balanced package of incentives (both financial and non-financial) may also increase health promoters’ motivation [46, 47]. In our study, health promoters did not receive any extra financial incentive, but they were paid through a social plan. Our results are similar to those reported by Curotto et al. [48] that showed that the possibility of having an active role in cervical cancer prevention activities was a main motivation factor for health promoters who were paid staff of the health system.

Understanding the economic and political context in which complex interventions are implemented is key for the analysis of implementation fidelity [10, 22, 49]. The health system in which interventions take place, its general human resource policies and financing, define the space in which interventions can operate regarding incentives, working conditions, and career perspective [49]. Unlike health promoters in Jujuy, in La Matanza, health promoters were paid through a social plan but did not belong to the permanent staff of the health system. This fact resulted in unstable or unsustainable financing, and consequently, a reduction of the number of health promoters that were involved in cervical cancer prevention. Lack of adequate financing is one of the major obstacles that is keeping health promoter programs from reaching their full potential [50]. A recent systematic review showed that lack of financing was the most common challenge (together with the lack of supplies) facing 29 national health promoter programs in LMIC [45, 51]. In our study, lack of funding had a negative impact on fidelity because it reduced the availability of trained human resources and the frequency of the offer of self-collection”.

Incorporating research into implementation, and political will, has been identified as factors associated with faster diffusion of an innovation and its sustainability [52, 53]. Our research project was carried out in a programmatic context and run collaboratively between CEDES (an NGO with broad experience in public health research), the NPCCP, and municipal health authorities and staff. In La Matanza, stakeholders participated in the plan, implementation, and fidelity evaluation of the strategy. Thus, this study can serve as a model of how implementation science can be used to plan, conduct, and evaluate strategies implemented on a large scale in programmatic, real-world contexts. The evaluation of implementation fidelity of complex strategies in real-world contexts is crucial to identify factors that limit or facilitate their scaling-up. It is particularly important in low- and middle-resource settings, which need to optimize resources to benefit as many people as possible and improve public health.

Our study has some limitations that should be considered. Observations might bias results due to the influence that the presence of a researcher (an outsider) may produce during the observation. Thus, results regarding the offer of self-collection might not reflect how it is usually offered in the absence of the observers. Despite this limitation, observations are one of the most widely used techniques for content evaluation in fidelity studies, because they are more appropriate than interviews in which participants only self-report and allow a deep description of the implementation context [10, 30]. In our study, to minimize observation bias, we included female observers with no link with the local health system, who were trained so as not to interfere during the offering of self-collection. In addition, health promoters were explained that observations were not an evaluation of their performance, but a way to understand how self-collection was locally offered. Health promoters were also reassured about anonymity of data collected during the observations.

## Conclusion

HPV self-collection has great potential for cervical cancer prevention, with a concrete possibility of saving thousands of lives in LMIC. Implementation science tools and frameworks are essential to enhance the way HPV-based strategies in cervical cancer prevention are implemented and evaluated. Our case study shows how HPV self-collection in programmatic contexts can be strengthened by using frameworks and tools used by Implementation Science. Specifically, it shows how the analysis of fidelity and adaptations of HPV self-collection in real-world contexts are key to measure and maximize its effectiveness in LMIC.

## Supplementary Information

**Additional file 1.** Standards for Reporting Implementation Studies: the StaRI checklist for completion.

**Additional file 2.** Leaflet.

**Additional file 3.** Adherence to the core components of EMA strategy and moderating factors.

**Additional file 4.** Submission explanation.

## Data Availability

The datasets used and/or analyzed during the current study are available from the corresponding author on reasonable request.

## Abbreviations

ASCUS+: Atypical squamous cells of undetermined significance or worse (Include: atypical squamous cells of undetermined significance atypical cells cannot rule out high-grade squamous intraepithelial lesion (ASC-H); low/high-grade squamous intraepithelial lesions (LSIL /HSIL); and cancer)
CFIF: Conceptual Framework for Implementation Fidelity
CHWs: Community health workers
HPV: Human papillomavirus
JDP: Jujuy demonstration project
NCCPP: National Cervical Cancer Prevention Program
SITAM: National Screening Information System (for its initials in Spanish)
WHO: World Health Organization

## References

[1] Das M (2021). WHO launches strategy to accelerate elimination of cervical cancer. Lancet Oncol.

[2] Arbyn M, Smith SB, Temin S, Sultana F, Castle P, Collaboration on Self-Sampling and HPV Testing (2018). Detecting cervical precancer and reaching underscreened women by using HPV testing on self samples: updated meta-analyses. BMJ..

[3] Arrossi S, Thouyaret L, Herrero R (2015). Effect of self-collection of HPV DNA offered by community health workers at home visits on uptake of screening for cervical cancer (the EMA study): a population-based cluster-randomised trial. Lancet Glob Health.

[4] Gök M, Heideman DA, van Kemenade FJ, Berkhof J, Rozendaal L, Spruyt JW, et al. HPV testing on self-collected cervicovaginal lavage specimens as screening method for women who do not attend cervical screening: cohort study. BMJ. 2010. 10.1136/bmj.c1040. 10.1136/bmj.c1040 PMC2837143 20223872

[5] Lazcano-Ponce E, Lorincz AT, Cruz-Valdez A, Salmerón J, Uribe P, Velasco-Mondragón E, et al. Self-collection of vaginal specimens for human papillomavirus testing in cervical cancer prevention (MARCH): a community-based randomised controlled trial. Lancet. 2011. 10.1016/S0140-6736(11)61522-5. 10.1016/S0140-6736(11)61522-5 22051739

[6] Arbyn M, Verdoodt F, Snijders PJ, Suonio E, Dillner L, et al. Accuracy of human papillomavirus testing on self-collected versus clinician-collected samples: a meta-analysis. Lancet Oncol. 2014. 10.1016/S1470-2045(13)70570-9. 10.1016/S1470-2045(13)70570-9 24433684

[7] Giorgi Rossi P, Marsili LM, Camilloni L, Iossa A, Lattanzi A, Sani C, et al. The effect of self-sampled HPV testing on participation to cervical cancer screening in Italy: a randomised controlled trial (ISRCTN96071600). Br J Cancer. 2011. 10.1038/sj.bjc.6606040. 10.1038/sj.bjc.6606040 PMC3031894 21179038

[8] Arrossi S, Paolino M, Thouyaret L, Laudi R, Campanera A (2017). Evaluation of scaling-up of HPV self-collection offered by community health workers at home visits to increase screening among socially vulnerable under-screened women in Jujuy Province, Argentina. Implement Sci.

[9] Carroll C, Patterson M, Wood S, Booth A, Rick J, Balain S (2007). A conceptual framework for implementation fidelity. Implement Sci.

[10] Hasson H, Blomberg S, Dunér A (2012). Fidelity and moderating factors in complex interventions: a case study of a continuum of care program for frail elderly people in health and social care. Implement Sci.

[11] Allen J, Linnan L, Emmons K, Browson R, Graham C, Proctor E (2012). Fidelity and its relationship to implementation effectiveness, adaptation, and dissemination. Dissemination and Implementation Research in Health.

[12] Pinnock H, Barwick M, Carpenter C, Eldridge S, Grandes G, Griffiths CJ (2017). Standards for Reporting Implementation Studies (StaRI) statement. BMJ.

[13] Morse JM, Tashakkori A, Teddie C (2003). Principles of mixed methods and multimethod research design. Handbook of Mixed Methods in Social & Behavioral Research.

[14] Hogue A, Henderson CE, Dauber S, Barajas PC, Fried A, Liddle HA (2008). Treatment adherence, competence, and outcome in individual and family therapy for adolescent behavior problems. J Consult Clin Psychol.

[15] Spillane V, Byrne MC, Byrne M, Leathem CS, O'Malley M, Cupples ME (2007). Monitoring treatment fidelity in a randomized controlled trial of a complex intervention. J Adv Nurs.

[16] Bellg AJ, Borrelli B, Resnick B, Hecht J, Minicucci DS, Ory M (2004). Treatment Fidelity Workgroup of the NIH Behavior Change Consortium. Enhancing treatment fidelity in health behavior change studies: best practices and recommendations from the NIH Behavior Change Consortium. Health Psychol.

[17] Cohen DJ, Crabtree BF, Etz RS, Balasubramanian BA, Donahue KE, Leviton LC (2008). Fidelity versus flexibility: translating evidence-based research into practice. Am J Prev Med.

[18] Santacroce SJ, Maccarelli LM, Grey M (2004). Intervention fidelity. Nurs Res.

[19] Siedlecki SL (2018). Research intervention fidelity: tips to improve internal validity of your intervention studies. Clin Nurse Spec.

[20] Dusenbury L, Brannigan R, Falco M, Hansen W (2003). A review of research on fidelity of implementation: implications for drug abuse prevention in school settings. Health Educ Res.

[21] Fixen DL, Naoon SF, Blase KA (2005). Implementation research: a synthesis of the literature.

[22] Hasson H, Blomberg S, Dunér A (2012). Fidelity and moderating factors in complex interventions: a case study of a continuum of care program for frail elderly people in health and social care. Implement Sci.

[23] Arrossi S, Paolino M, Laudi R, Gago J, Campanera A, Marín O (2019). Programmatic human papillomavirus testing in cervical cancer prevention in the Jujuy Demonstration Project in Argentina: a population-based, before-and-after retrospective cohort study. Lancet Glob Health.

[24] Craig P, Dieppe P, Macintyre S, Michie S, Nazareth I, Petticrew M (2008). Developing and evaluating complex interventions: the new Medical Research Council guidance. BMJ..

[25] Proctor EK, Powell BJ, McMillen JC (2013). Implementation strategies: recommendations for specifying and reporting. Implement Sci.

[26] Taplin SH, Rodgers AB (2010). Toward improving the quality of cancer care: addressing the interfaces of primary and oncology-related subspecialty care. J Natl Cancer Inst Monogr.

[27] Nekhlyudov L, Latosinsky S (2010). The interface of primary and oncology specialty care: from symptoms to diagnosis. J Natl Cancer Inst Monogr.

[28] Arrossi S, Thouyaret L, Paul L (2015). Recomendaciones para el tamizaje, seguimiento y tratamiento de mujeres para la prevención del cáncer cervico-uterino en el marco de la incorporación de la prueba de VPH.

[29] Davies L, LeClair KL, Bagley P (2020). Face-to-face compared with online collected accounts of health and illness experiences: a scoping review. Qual Health Res.

[30] Pérez MC, Chandra D, Koné G, Singh R, Ridde V, Sylvestre MP (2020). Implementation fidelity and acceptability of an intervention to improve vaccination uptake and child health in rural India: a mixed methods evaluation of a pilot cluster randomized controlled trial. Implement Sci Commun.

[31] Gale NK (2013). Using the framework method for the analysis of qualitative data in multi-disciplinary health research. BMC Med Res Methodol.

[32] Braun V, Clarke V (2006). Using thematic analysis in psychology. Qual Res Psychol.

[33] Curotto M, Zalacaín-Colombo J, Paolino M, Arrossi S (2018). Adopción e implementación del ofrecimiento de la autotoma VPH por agentes sanitarios en Jujuy, Argentina [Adoption and implementation of HPV self-collection sampling by CHWs in Jujuy, Argentina]. Salud Publica Mex.

[34] von Thiele SU, Aarons GA, Hasson H (2019). The value equation: three complementary propositions for reconciling fidelity and adaptation in evidence-based practice implementation. BMC Health Serv Res.

[35] Sundell K, Beelmann A, Hasson H, von Thiele SU (2016). Novel programs, international adoptions, or contextual adaptations? Meta-analytical results from German and Swedish Intervention Research. J Clin Child Adolesc Psychol.

[36] Sanchez Antelo V, Szwarc L, Paolino M, Saimovici D, Massaccesi S, Viswanath K, Arrossi S. A Counseling Mobile App to Reduce the Psychosocial Impact of Human Papillomavirus Testing: Formative Research Using a User-Centered Design Approach in a Low-Middle-Income Setting in Argentina. JMIR Form Res. 2022;6(1):e32610. 10.2196/32610. 10.2196/32610 PMC8796044 35023843

[37] Paolino M, Campanera A, Martiarena S (2019). Adherencia al triaje en contexto de tamizaje con autotoma del test de virus del papiloma humano en la provincia de. Jujuy Rev Argent Salud Pública.

[38] Paolino M, Gago J, Pera AL, Cinto O, Thouyaret L, Arrossi S (2020). Adherence to triage among women with HPV-positive self-collection: a study in a middle-low income population in Argentina. Ecancermedicalscience.

[39] Sancho-Garnier H, Tamalet C, Halfon P (2013). HPV self-sampling or the Pap-smear: a randomized study among cervical screen- ing non-attenders from lower socioeconomic groups in France. Int J Cancer.

[40] Holme F, Maldonado F, Martinez-Granera OB (2020). HPV-based cervical cancer screening in Nicaragua: from testing to treat- ment. BMC Public Health.

[41] Giorgi Rossi P, Fortunato C, Barbarino P (2015). Self-sampling to increase participation in cervical cancer screening: an RCT comparing home mailing, distribution in pharmacies, and recall letter. Br J Cancer.

[42] Paolino M, Sankaranarayanan R, Arrossi S (2013). Determinantes sociales del abandono del diagnóstico y el tratamiento de mujeres con Papanicolaou anormal en Buenos Aires. Argentina Rev Pan de Salud Pública.

[43] Zapka J, Taplin S, Anhang Price R (2010). Factors in quality care—the case of follow-up to abnormal cancer screening tests— problems in the steps and interfaces of care. J Natl Cancer Inst Monogr.

[44] Arrossi S, Paolino M, Sanchez Antelo V, Thouyaret L, Kohler E, Cuberli M, et al. Effectiveness of an mHealth intervention to increase adherence to triage of HPV DNA positive women who have performed self-collection (the ATICA study): a hybrid type I cluster randomized effectiveness-implementation trial. Lancet Reg HealthAmericas. 2022;9. 10.1016/j.lana.2022.100199. 10.1016/j.lana.2022.100199 PMC9159703 35655914

[45] Perry H, Crigler L, Kok M, Ballard M, Musoke D, LeBan K, et al. Community Health Worker Programs at the Dawn of a New Era: 11. Leading the Way to Health for All. BMC. Health Res Policy Syst. 2021. 10.1186/s12961-021-00755-5. 10.1186/s12961-021-00755-5 PMC8506098 34641891

[46] Naimoli JF, Perry HB, Townsend JW, Frymus DE, McCaffery JA (2015). Strategic partnering to improve community health worker programming and performance: features of a community-health system integrated approach. Hum Resour Health.

[47] Colvin CJ, Hodgins S, Perry HB (2021). Community health workers at the dawn of a new era: 8. Incentives and remuneration. Health Res Policy Syst.

[48] Curotto M, Barletta P, Paolino M, Arrossi S (2017). La perspectiva de los agentes sanitarios sobre la incorporación programática de la autotoma del test de VPH. Cad Saude Publica.

[49] Kok MC, Kane SS, Tulloch O, Ormel H, Theobald S, Dieleman M (2015). How does context influence performance of community health workers in low- and middle-income countries? Evidence from the literature. Health Res Policy Syst.

[50] Masis L, Gichaga A, Zerayacob T, Lu C, Perry HB (2021). Community health workers at the dawn of a new era: 4. Programme financing. Health Res Policy Syst.

[51] Health for the People: National Community Health Programs from Afghanistan to Zimbabwe. https://chwcentral.org/wp-content/uploads/2021/11/Health_for_the_People_Natl_Case%20Studies_Oct2021.pdf. Accessed 23 Sep 2022.

[52] Khresheh R, Lesley B. Practice–research engagement. Jordanian experience in three Ministry of Health hospitals. Action Res. 2007. 10.1177/1476750307077313.

[53] Yamey G. Scaling up global health interventions: a proposed framework for success. PLoS Med. 2011. 10.1371/journal.pmed.1001049. 10.1371/journal.pmed.1001049 PMC3125181 21738450

