# Supplementary material for: Assessing the implementation fidelity of HPV self-collection offered by community health workers during home visits (the EMA strategy): a case study in a low-middle-resource setting in Argentina
Source: Implement Sci Commun. 2023 Jan 12;4:4. doi: 10.1186/s43058-022-00367-2 (PMC9836335; doi:10.1186/s43058-022-00367-2)
Supplement: Supplementary file 3 — Additional file 3. Adherence to the core components of EMA strategy and moderating factors. [file 43058_2022_367_MOESM3_ESM.docx]

**Additional file 3. Adherence to the core components of EMA strategy and moderating factors**

| **Adherence** | **INDICATORS** | **VALUE** | **Moderating factor**  **that affect level of adherence**  **(+ positive / - negative)** |
| --- | --- | --- | --- |
| **Content: core components of the strategy** | | |  |
| **TRAINING** | % of planned workshops implemented in La Mataza | 100% (n=2/2) | **Context:** Stakeholder engagement, Political will (+)  **Participant responsiveness:** motivation of health promoters, active participation of local stakeholders during planning and implementation of training (pre-implementation phase) (+) |
|  | % of HPs that participated in the two workshops | 95% (n=163/171) |  |
|  | % of HPs with adequate knowledge about the EMA strategy | 92% (n=157/171) |  |
| **OFFER OF HPV SELF-COLLECTION** | % of SC offered during home visit (following EMA model) | 54% (n=40/74) | **Context:** Urban Insecurity (-) / reduction of number of health promoters that were involved in cervical cancer prevention (-)  Previous experience in Community work allows for adaptation related place of offer (+) |
|  | ***Identification of target population (among all women, n=74)*** |  |  |
|  | % of SC offers in which HPs ask the woman age | 70% (n=52/74) | **Context:** Less time to the offer (-) /reduction of information during the offer  **Intervention complexity:** several pieces of information |
|  | % of SC offers which HPs ask if the woman was pregnant | 34% (n=25/74) |  |
|  | % of SC offers in which HPs ask if the woman had a previous HPV test | 22% (n=16/74) |  |
|  | **Key information during SC offer (among women considered eligible, n=63)** | |  |
|  | % of SC offers in which HPs mentioned information about HPV test | 43% (n=27/63) |  |
|  | % of SC offers in which HPs mentioned information about CC prevention | 68% (n=43/63) |  |
|  | % of SC offers in which HPs mentioned information about SC | 47% (n=29/63) |  |
|  | % of SC offers in which HPs mentioned that SC is painless? | 27% (n=17/63) |  |
|  | % of SC offers in which HPs mentioned information about possible HPV results | 41% (n=26/63) |  |
|  | % of SC offers in which HPs mentioned information about results delivery date | 51% (n=32/63) |  |
|  | % of SC offers in which HPs mentioned information about results delivery methods | 82% (52/63) |  |
|  | **Explanation on how to perform self-collection (among women who accept SC, n=50)** | |  |
|  | % of SC offers in which HPs suggest a private place to perform SC | 78% (n=39/50) |  |
|  | % of SC offers in which HPs mentioned different positions to perform SC. | 36% (n=18/50) |  |
|  | % of SC offers in which HPs warned women that should be careful with the liquid inside the tube | 54% (n=27/50) |  |
|  | % of SC offers in which HPs explained to the women that they had to insert the brush into their vagina until it reaches the bottom | 70% (35/50) |  |
|  | % of SC offers in which HPs explained that woman had to rotate the brush 3 times. | 90% (45/50) |  |
|  | % of SC offers in which HPs used communication support material | 48% (n=24/50) |  |
| **SAMPLE HANDLING AND TRANSPORTATION** | *% of SC offers in which HPs check that the tube was correctly closed* | 96% (n=48/50) | **Facilitator strategies:** Feedback and supervision in different levels of the health system: health care centers – HPV lab– second-level hospitals  **Participant responsiveness:** motivation and active participation of health promoters, supervisors and local stakeholder |
|  | % of SC offers in which HPs transported the tube in vertical position | 96% (n=48/50) |  |
|  | % of SC offers in which HPs labeled collectors | 98% (n=49/50) |  |
|  | % of SC offers in which HPs filled HPV-form | 96% (n=48/50) |  |
| **FOLLOW UP AND TREATMENT** | % of HPV positive women with triage Pap at 120 days | 38% (318/830) | **Intervention complexity:** different levels of health system involved in screening – triage – diagnosis and treatment (-) |
|  | % of HPV-positive/abnormal Pap women with colposcopy | 64% (n=34/49) |  |
|  | % of CIN2+ with treatment | 100% (n=13/13) |  |
| **Dose** | | |  |
| Duration | Mean duration of SC offers | 8 minutes |  |
| **Coverage** | | |  |
| Coverage | % of women who accepted SC | 79% (50/63) |  |
